# Modeling Experimental Parameters for the Fabrication of Multifunctional Surfaces Composed of Electrospun PCL/ZnO-NPs Nanofibers

**DOI:** 10.3390/polym13244312

**Published:** 2021-12-09

**Authors:** Pedro J. Rivero, Juan P. Fuertes, Adrián Vicente, Álvaro Mata, José F. Palacio, María Monteserín, Rafael Rodríguez

**Affiliations:** 1 Engineering Department, Public University of Navarre, Campus Arrosadía S/N, 31006 Pamplona, Spain; juanpablo.fuertes@unavarra.es (J.P.F.); adrian.vicente@unavarra.es (A.V.); mata.116057@e.unavarra.es (Á.M.); rafael.rodriguez@unavarra.es (R.R.); 2 Institute for Advanced Materials and Mathematics (INAMAT^2^), Public University of Navarre, Campus Arrosadía S/N, 31006 Pamplona, Spain; 3 Centre of Advanced Surface Engineering, AIN, 31191 Cordovilla, Spain; jfpalacio@ain.es (J.F.P.); mmonteserin@ain.es (M.M.)

**Keywords:** PCL, electrospinning, ZnO nanoparticles, DoE, wettability, corrosion rate

## Abstract

In this work, a one-step electrospinning technique has been implemented for the design and development of functional surfaces with a desired morphology in terms of wettability and corrosion resistance by using polycaprolactone (PCL) and zinc oxide nanoparticles (ZnO NPs). The surface morphology has been characterized by confocal microscopy, scanning electron microscopy (SEM), atomic force microscopy (AFM) and water contact angle (WCA), whereas the corrosion resistance has been evaluated by Tafel polarization curves. Strict control over the input operational parameters (applied voltage, feeding rate, distance tip to collector), PCL solution concentration and amount of ZnO NPs have been analyzed in depth by showing their key role in the final surface properties. With this goal in mind, a design of experiment (DoE) has been performed in order to evaluate the optimal coating morphology in terms of fiber diameter, surface roughness (Ra), water contact angle (WCA) and corrosion rate. It has been demonstrated that the solution concentration has a significant effect on the resultant electrospun structure obtained on the collector with the formation of beaded fibers with a higher WCA value in comparison with uniform bead-free fibers (dry polymer deposition or fiber-merging aspect). In addition, the presence of ZnO NPs distributed within the electrospun fibers also plays a key role in corrosion resistance, although it also leads to a decrease in the WCA. Finally, this is the first time that an exhaustive analysis by using DoE has been evaluated for PCL/ZnO electrospun fibers with the aim to optimize the surface morphology with the better performance in terms of corrosion resistance and wettability.

## 1. Introduction

The design of superhydrophobic surfaces with water contact angles higher than 150° has been object of a great scientific and industrial interest in fields as diverse as self-cleaning [1], oil-water separation [2,3], antifouling [4], ice repellency [5,6,7], or corrosion-resistant surfaces [8,9,10], among others. The inspiration of this high water repellency behavior originated from natural surfaces (i.e., lotus leaf, duck feather, butterfly wing) [11,12,13], and numerous works are focused on the development of functional surfaces with this special wettability thanks to the cooperation between the chemical composition (low surface free energy) and hierarchical structure (micro- and nanostructures) [14,15]. In order to create these functionalized surfaces with a hierarchical structure, multiple approaches have been developed to manufacture rougher surfaces by using chemical vapor deposition [16,17], laser texturing [18,19], laser-assisted magnetron sputtering [20] or chemical etching [21]. All these methods have shown a great degree of effectiveness and durability, although expensive machinery and a precise control over the experimental conditions are required. However, one of the deposition techniques which is gaining more attention in the scientific community due to its easy control, simplicity and versatility in fabricating hierarchical structures is electrospinning [21,22]. The basic fiber-forming process taking place in electrospinning is controlled by electric charges located on the jet and by their interactions with an externally applied field, achieving fibers with a predetermined surface topology and a variable fiber diameter [23]. The fibers are formed thanks to the application of a high voltage to a liquid polymeric precursor which is held at the end of a conductive capillary tube (needle) and upon the action of the electric field, the droplets are gradually elongated, forming a characteristic conical shape on the tip of the needle which is denoted as “Taylor cone” [24]. According to this, multiple parameters have to be perfectly controlled in order to obtain fibers with the desired morphology, being the most representative the nature of the polymeric precursor (i.e., concentration, molecular weight, solvent viscosity, surface tension) and the experimental operational conditions (i.e., feeding rate, applied voltage, distance tip-collector, type of collector) [25,26,27]. In addition, the relative humidity (%RH) is also considered a key parameter in the resultant fiber morphology and chain conformation as well as in the wettability and mechanical properties [28,29]. Several works have demonstrated that smooth fibers are obtained at low RH values, whereas wrinkly and grooved texture, pore formation or even surface features are observed at high RH values [30,31,32]. Another important aspect to remark upon is that the use of electrospun nanofibrous coatings onto metallic substrates can be considered as a novel approach for improving the resultant corrosion resistance [33]. Among all the polymeric precursors, polycaprolactone (PCL) polymer can be used as a promoting biomaterial due to its intrinsic characteristics such as nontoxicity, robust mechanical properties, biocompatibility and excellent biodegradability [34], showing at the same time a highly hydrophobic behavior [35]. In addition, the incorporation of metallic oxide nanoparticles into the electrospun fibers can offer additional advantages, enhancing the polymeric coating properties associated to the electrospun fibers. The use of zinc oxide nanoparticles (ZnO) is of great interest in biomedical applications due to its intrinsic properties such as antibacterial, non-toxicity and anticancer behavior [36,37,38]. In fact, previous works have reported the fabrication of electrospun polycaprolactone membranes incorporated with ZnO NPs (PCL/ZnO) as skin substitutes with enhanced fibroblast proliferation and wound healing [39], or even an improvement of both angiogenic and cell adhesion properties of electrospun PCL tissue engineering [40]. In these works, the existence of an optimal concentration to induce inflammation and inhibit bacterial growth has been observed. In addition, the ZnO concentration has a strong effect on the resultant fiber morphology because it is well known that during the electrospinning process the overall tension in the fibers depends on the self-repulsion of the excess charges on the jet which is a parameter to control the deposition process [41]. However, it is important to remark that the use of ZnO NPs can be extrapolated to other research lines such as the design of anticorrosion surfaces [42].

In this work, the incorporation of zinc oxide (ZnO) nanoparticles into a PCL polymeric precursor is evaluated by using a design of experiment (DoE) statistical tool in order to understand the influence of the fiber generation parameters on its most representative factors. On the one hand, the main factors that have been analyzed are PCL concentration, tip-collector distance, applied voltage, feeding rate, and presence/absence of ZnO. On the other hand, factors that have been studied are the fiber size, contact angle (to analyze the wettability), surface roughness, presence of beads and the corrosion rate. Finally, it is the first time that the DoE methodology has been evaluated over PCL/ZnO electrospun fibers in terms of wettability and corrosion resistance for a further implementation in biomedical or industrial applications.

## 2. Experimental Section

### 2.1. Materials and Reagents

In this work, polycaprolactone (PCL) (Mn = 80,000 g/mol), zinc oxide nanoparticles (ZnO NPs) (nanopowder, <100 nm particle size), *N*,*N*-dymethylformamide (DMF) (anhydrous, 99.8%) and dichloromethane (DCM) (ACS reagent, ≥99.5%) were provided from Sigma Aldrich (St. Louis, MO, USA). All the materials required in the experiment were used without any further purification. Firstly, the electrospinning coatings have been deposited on standard glass slides in order to analyze the morphology and the wettability. Secondly, the corrosion behavior of these coatings has been evaluated by using aluminum alloy substrates (AA6061T6) in 3.5 wt% NaCl (M = 58.44 g/mol, PanReac AppliChem, Barcelona, Spain) as corrosive medium.

### 2.2. Electrospinning Procedure

In this work, PCL was dissolved in a mixture of DCM and DMF in a ratio 7:3 under stirring (200 rpm) for 12 h. The solutions were pumped into a 5 mL syringe and placed on the syringe pump. The positive electrode for all the depositions was a 20-gauge needle with an inner diameter of 0.6 mm and the spinning process lasted 15 min for each sample. All the experiments were conducted at room temperature with a relative humidity (RH) of 35–40%, respectively. A summary of the experimental conditions for the fabrication of the electrospun samples is presented in Table 1 as a function of the polymer concentration, ZnO NPs proportion, flow rate, applied voltage and nozzle-to-collector distance.

### 2.3. Characterization Techniques

A stress-controlled rheometer (DHR-1; TA Instruments) equipped with a Peltier temperature system for temperature control and concentric cylinder measuring system was used to measure the rheological property. Briefly, 25 mL sample was loaded to the cup of concentric cylinder measuring system. The sample was further exposed to 300 s of equilibration at 25 °C. Then, a flow sweep was carried out, measuring viscosity and stress at shear rates between 1 × 10^−3^ and 1000 s^−1^.

The surface morphology and the thickness of the whole electrospun samples has been evaluated by using a confocal microscope (model S-mart, SENSOFAR METROLOGY, Barcelona, Spain). The confocal microscope applies a course shift single algorithm with an objective of EPI 50X v35 for an area of 340.03 × 283.73 µm. According to the standard ISO 25178, the roughness surface (S-L) measurements have been obtained with three different filters: a low filter (F-operator-level), a high filter (S-filter, standard cut off λ_s_: 2.5 µm) and a Gaussian filter (L-filter, standard cut off λ_c_: 0.08 mm, Sa < 0.02 µm). These topography measurements were used for estimating the average fiber diameter (Df) and roughness surface (Sa) of the samples.

The water contact angle (WCA) measurements were carried out with a CAM 100 contact angle goniometer (CAM 100, KSV Instruments, Burlington, VT, USA) using distilled water. The static water contact angle was measured three times and in six different places of the sample. The results were calculated with the average and their average deviations of the measurements.

**Figure 1:**
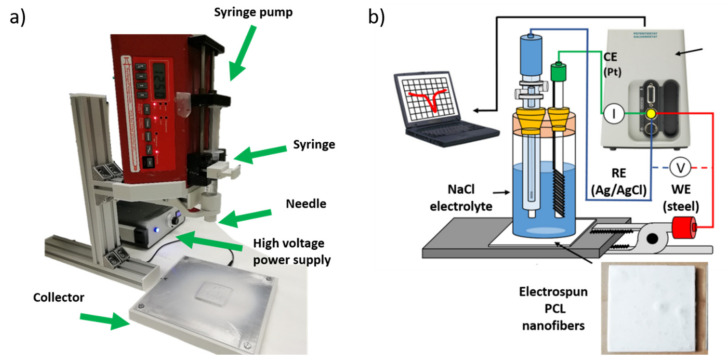
(**a**) Schematic representation of the electrospinning setup used for the fabrication of the electrospun samples; (**b**) setup used for the corrosion test in the Tafel curves.

Finally, the electrochemical measurements of Tafel polarization curves were carried out on an Autolab Potentiostat/Galvanostat PGSTAT302N (Metrohm, Herisau, Switzerland). All corrosion tests were performed at room temperature in 3.5 wt% NaCl aqueous solutions, using a conventional three-electrode cell consisting of a working electrode (steel AISI 304), a silver chloride Ag-AgCl reference electrode and a platinum counter electrode. Before conducting all the experiments, the samples were immersed in 3.5 wt% NaCl electrolyte for 30 min to make sure that the system is in steady state and with the open circuit potential (OCP) stabilized. A schematic representation of the electrospinning setup as well as the electrochemical cell for the corrosion tests performed on the electrospun PCL samples is summarized in Figure 1.

The Tafel polarization measurements were obtained by scanning the electrode potential automatically from −200 mV to +200 mV with respect to the OCP voltage at a scan rate of 1.5 mV.s^−1^.

The output from these experiments yielded a polarization curve of the current density vs. the applied potential. The resulting corrosion current can be calculated by using Tafel slope analysis, where a relationship is established between the current density and the electrode potential during the polarization test. The corrosion rate (CR expressed in mm/year) is calculated according to the following equation [43,44,45]:

(1) $$\mathrm{CR}=\frac{j_{\mathrm{corr}}\;M}{V\cdot D}\times 3270$$

where j_corr_ is the current density (A.cm^−2^), M is the atomic mass of aluminum alloy, V is the valence (number of electrons that are lost during the oxidation reaction), D is the density in grams/cm^3^, respectively.

### 2.4. Evolution of Raw Data Using Design of Experiments

The employed technique of design of experiment (DoE) has been based on a central composite design (CCD) with two central points and 10 face-center points (a total of 44 experiments). The studied factors (PCL concentration, distance, voltage, flow rate and amount of ZnO denoted as inhibitor presence) are shown in Table 2, with their corresponding low and high levels. Likewise, the response variables analyzed were the fiber diameter, water contact angle, surface roughness, the presence of fiber beads and corrosion resistance, respectively.

## 3. Results and Discussion

### 3.1. Surface Morphology

**Figure 2:**
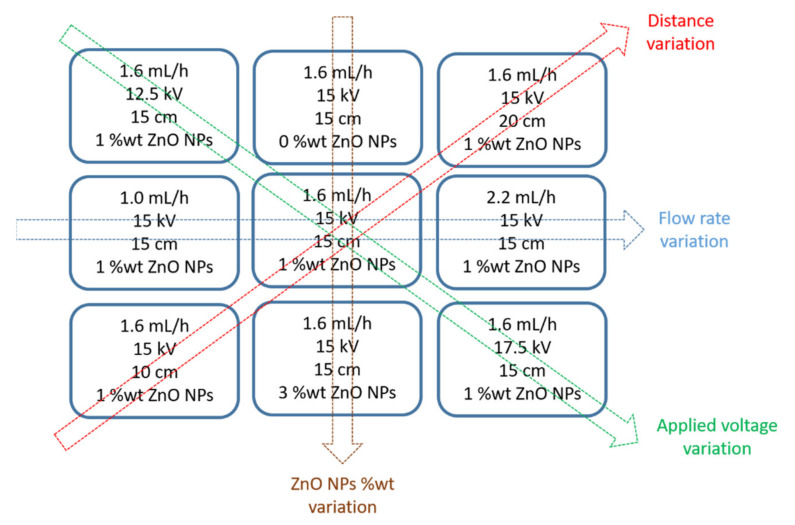
Summary of all the electrospun coatings as a function of the variation of the experimental conditions (flow rate, distance, applied voltage and ZnO NPs wt%) for a fixed PCL concentration (10 wt%), respectively.

The first step has been to analyze the effect of the input operational parameters (flow rate, applied voltage, tip to collector distance and ZnO NPs wt%) in the fiber morphology of the coatings. In Figure 2, a summary of the experimental details for the fabrication of the electrospun fibers at a fixed polymeric concentration (10 wt%) is shown. In addition,

Initially, the lower and upper limit values of the electric potential were investigated in order to obtain a successful deposition of the resultant nanofibers. Voltage values lower than 10 kV were shown to be very low without the deposition of fibers in the collector, whereas values higher than 20 kV were shown to be higher in order to form the Taylor cone, showing the presence of fiber defects. The experimental results have demonstrated that the Taylor cone has been clearly formed in the range of 12.5–17.5 kV. According to this, these values have been selected to be the lower and upper limits of the applied voltage for the set of experiments of the parameter optimization process. Once this parameter has been optimized, the effect of varying the PCL concentration, ZnO NPs proportion, distance and flow rate for this applied voltage range was analyzed in the resultant fiber diameter as shown in Table 3.

**Figure 3:**
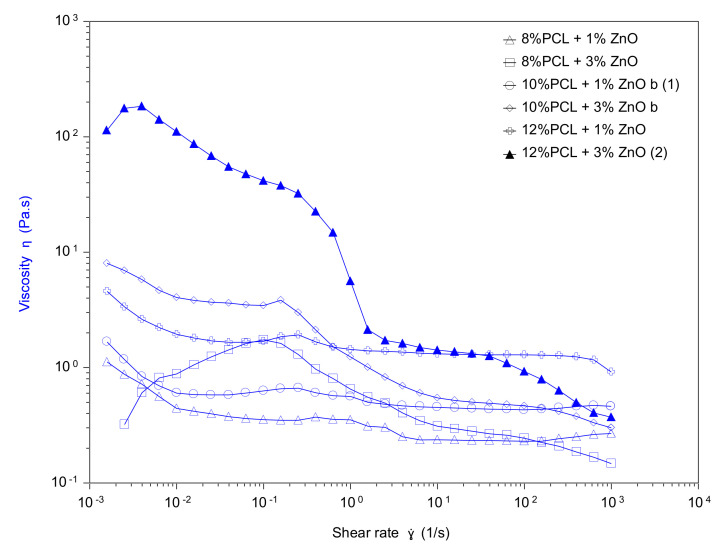
Viscosity of the PCL solutions with different contents of ZnO as a function of shear rate.

From these experimental results, it was demonstrated that the fiber diameter of the PCL electrospun samples showed a gradual decrease from 4.21 ± 0.13 µm (S2), 3.66 ± 0.21 µm (S3) up to 3.18 ± 0.11 µm (S4) when the applied voltage was increased from 12.5 up to 17.5 kV, respectively. This result, of obtaining narrower fibers with an increase in the applied voltage, is associated to the production of a higher electrostatic repulsive force on the fluid jet which is concordance with the bibliography [46,47,48]. Another interesting result is that an increase in the flow rate from 1000 µL/h up to 2200 µL/h produced an increase in the fiber diameter from 3.21 ± 0.11 µm (S1), 3.66 ± 0.21 µm (S3) up to 4.02 ± 0.13 µm (S5), respectively. This result was also in accordance with the literature because as the flow rate increased, the available polymer volume was higher, making possible an increase in the fiber diameter [49,50,51,52]. In addition, the effect of increasing the distance between the tip and collector resulted in the formation of thinner electrospun fibers from 3.95 ± 0.32 µm (10 cm, S6), 3.66 ± 0.21 µm (15 cm, S3) up to 3.32 ± 0.13 µm (20 cm, S7) which was in accordance with the literature [33,53]. In addition, the effect of the variation in the PCL solution concentration was also evaluated, showing an increase in the resultant fiber diameter for a higher solution concentration from 2.13 ± 0.09 µm (8 wt%, S10), 3.66 ± 0.21 µm (10 wt%, S3) up to 4.02 ± 0.13 µm (12 wt%, S11). This aspect related to a higher fiber diameter with increasing polymer concentration was associated to a power law relationship [54]. A similar finding has been reported by [55] which suggests that the increment of polymer concentration makes possible an enlargement of the fiber diameter due to the increase of viscosity and surface tension of the spinning solution. According to this, the experimental values corroborate that a gradual increase in the PCL concentration (at fixed 1% ZnO NPs) produce an increase in the viscosity value from 0.2681 Pa.m (8 %PCL, S10), 0.4615 Pa.m (10 %PCL, S13) up to 0.9146 Pa.m (12 %PCL, S11). In addition, the addition of a high amount of ZnO (3 wt%) has produced a decrease in the resultant viscosity value (see Figure 3). The effect of solution viscosity on the morphological structures of the electrospun fibers was investigated. A higher solution viscosity is related to the extent of polymer chain molecular entanglement which is typically increased with the concentration. As can be clearly observed, the increase in the PCL concentration results in an increase in the resultant viscosity [56]. In addition, during the electrospinning, a solution with low viscosity (i.e., low viscoelastic strength) cannot compete with the electrostatic and coulombic repulsive forces, resulting in jet breakage. However, jets of solutions with higher viscosities do not break, making possible the apparition of continuous fibers. In this work, the increase in the fiber diameter as a function of the increase of polymer concentration is due to the higher viscoelastic forces resisting the elongational stretching of the jet [57].

**Figure 4:**
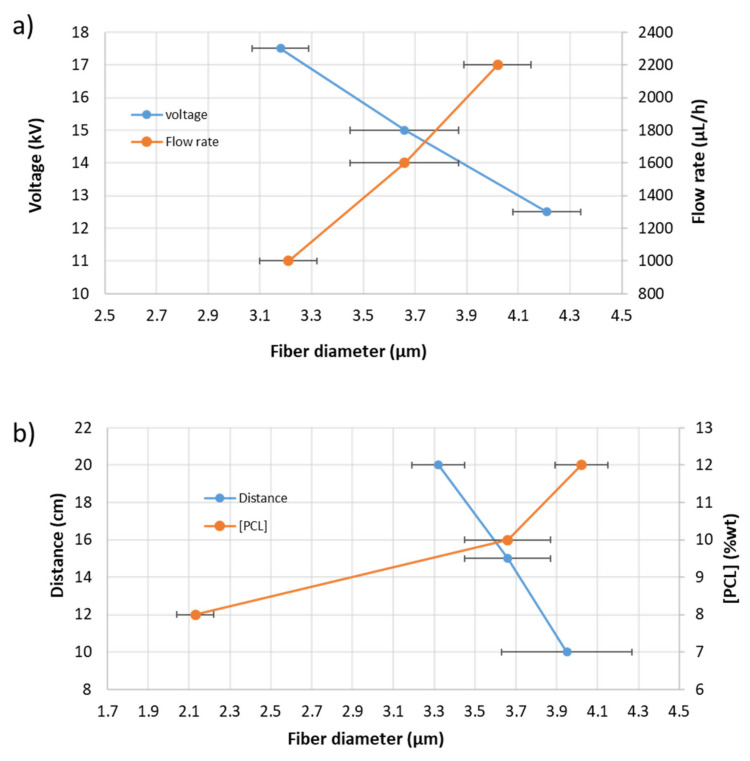
(**a**) Evolution of the fiber diameter as a function of the applied voltage and flow rate; (**b**) evolution of the fiber diameter as a function of distance and PCL solution concentration, respectively.

Figure 4, in order to have a better understanding of the influence of all these parameters in the fiber diameter morphology, shows the variation of the resultant fiber diameter as a function of the applied voltage and flow rate (Figure 4a) as well as a function of the distance tip to collector and PCL solution concentration (Figure 4b), respectively.

**Figure 5:**
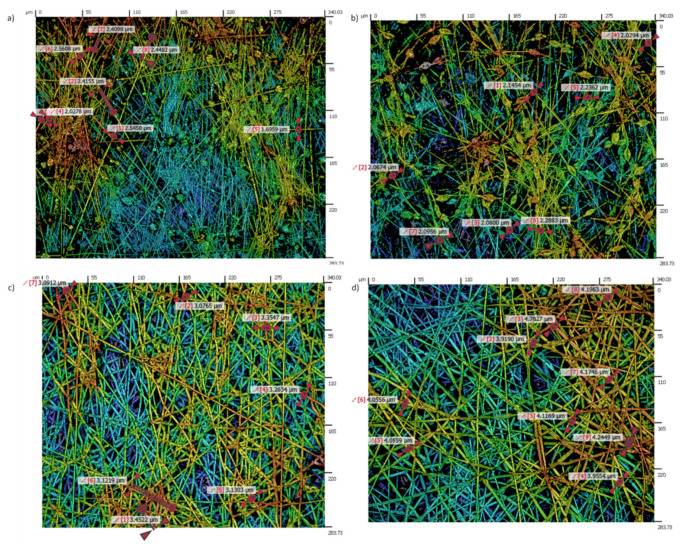
Evolution of the resultant surface morphology and fiber diameter for 8 wt% PCL with the presence of multiple beads (**a**,**b**); uniform interconnected fibers for 10 wt% PCL (**c**) and 12 wt% PCL (**d**), respectively.

An important point is that the solution concentration strongly influences the surface morphology because the most diluted PCL solution (8 wt%) has shown the presence of beads, whereas the most concentrated PCL solutions (10 or 12 wt%) have shown uniform fibers free of beads, as can be appreciated in the confocal images in Figure 4. In previous works it has been demonstrated that the solution concentration has a significant effect on the structure in the final polymer obtained on the collector [51]. A lower PCL concentration was found to have an insufficient viscosity to resist fiber deformation without defects at the applied electric field, being an ideal scenario for the beaded-fiber’s formation which is due to the high surface tension of the spinning fluid [30,55]. In Figure 5a,b can be clearly appreciated the formation of multiple beads (8 wt% PCL), whereas a completely fibrous interconnected structure with a web of sub-micron fibers can be observed for 10 and 12 wt% PCL, respectively. According to this, the complete transformation from beads to interconnected fibers begins at 10 wt% PCL and the average fiber diameter increases with the solution concentration (Figure 5c,d).

**Figure 6:**
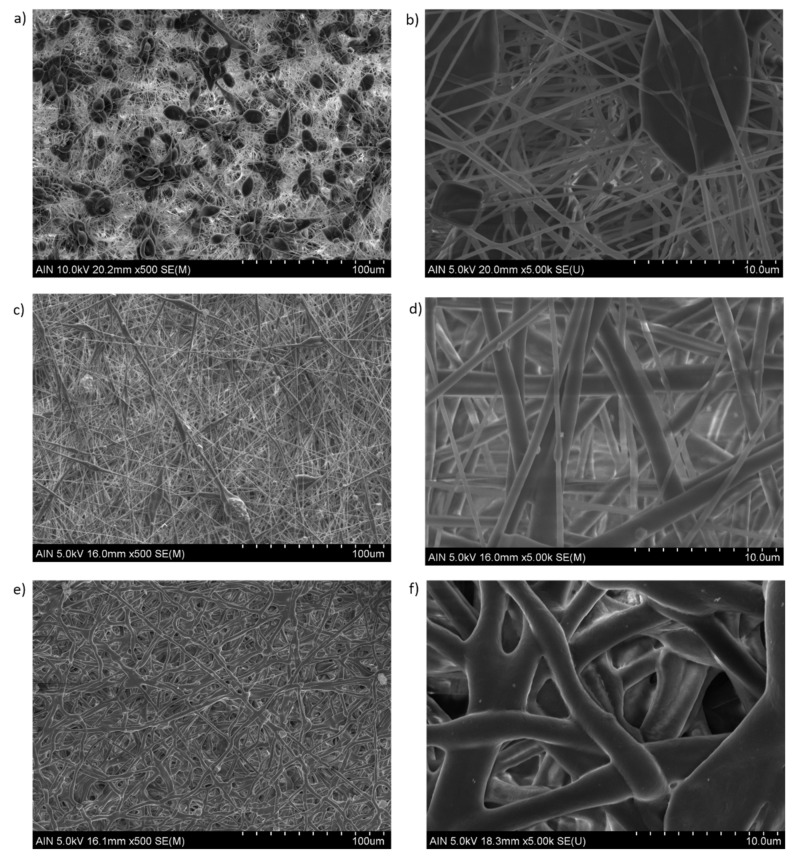
Scanning electron microscopy (SEM) of the electrospun fiber mats at different scale bars (100 and 10 µm). Fiber beaded formation at 8 wt% PCL (**a**,**b**). Total evaporation of the solvent with a dry polymer deposition onto the collector (**c**,**d**). Fiber merging with a flattening aspect when wet fibers impact onto the collector, showing an increase in the resultant fiber diameter (**e**,**f**).

In order to have a better appreciation of the surface morphology, Figure 6 shows SEM images of the samples at different PCL concentrations (8 and 12 wt%, respectively). First of all, as mentioned, the presence of multiple beads within the fibers can be appreciated for the sample composed of 8 wt% PCL (Figure 6a), showing the aspect of bead-on-string structures with oval shapes as can be observed in the magnified SEM image (Figure 6b). A higher polymer molecular weight (12 wt%) can generally stabilize the resultant fibrous mat structure, although different fiber morphology can be clearly appreciated as a function of the solvent evaporation. According to this, the formation of straight electrospun fibers with a round cross section (indicative of an elongational flow) are obtained when the resultant solvent has totally evaporated before reaching the collector (see Figure 6c,d). However, the formation of flat fibrous structures with wet fibers is observed when the solvent has not completely evaporated (see Figure 6e,f). These wet fibers can flatten upon impact onto the collector, showing a coalescence and as a result, the fiber diameter has increased [58]. Another aspect to mention is that by using electrospinning technique it is possible to modulate the formation of porous electrospun PCL fibers as a function of the selected solvent system [59,60,61]. After observing SEM images, the ratio used for the binary system (DCM:DMF) is adequate for the formation of smooth fibers for the PCL concentration range, showing only differences in the presence of beads or in the resultant fiber diameter, respectively.

Another aspect to mention is that the addition of ZnO NPs in the PCL solution has produced a slight change in the fiber diameter, as can be observed in S8 (3.68 ± 0.16 µm without the presence of ZnO NPs), S3 (3.66 ± 0.21 µm for 1 wt% ZnO NPs) and S9 (3.71 ± 0.11 µm for 3wt% NPs). A previous work has demonstrated that PCL-ZnO nanocomposites have shown a reduction in the resultant fiber diameter as the addition of ZnO NPs has increased up to 1 wt% concentration, this effect being more notorious for 0.4 wt% concentration [62]. This behavior of the fiber diameter reduction related to the addition of a specific amount of ZnO NPs is associated to a higher charge density on the surface of the ejected jet during the electrospinning process, resulting in more electric charges being carried by the electrospinning jet. As the charges carried by the jet increase, higher elongation forces are imposed on the jet under the electrical field, and as result, the diameter of the final fibers are narrower [63,64]. After observing Table 4, there is a similar tendency in the resultant average fiber diameter in the case of PCL/ZnO (1 wt%) in comparison with neat PCL fibers with a slight reduction in the fiber diameter. Once ZnO concentration overcomes a specific limit, the viscosity of the solution tends to increase which leads to the apparent increase in fiber diameter (PCL/ZnO 3 wt%). Despite the slight variation of the solution viscosity as a function of ZnO content, this parameter has no shown a significant effect on the average fiber diameter which has been also corroborated in previous works by using other different types of polymer such as polyvinyl chloride (PVC) [42] or poly(D,L-lactide) (PLA) [65].

**Figure 7:**
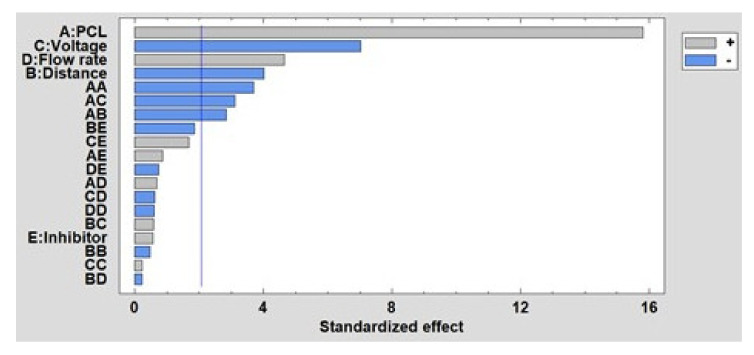
Standardized Pareto chart for fiber diameter.

Once the influence of the factors on the fiber diameter has been experimentally shown, the results obtained in the DOE is presented. In Figure 7, the Pareto diagram is shown, where the influence of the main parameters on the fiber diameter (PCL concentration, distance, voltage and presence of the inhibitor) is observed. Likewise, the Pareto diagram also shows the effect of the interactions between the main effects on the fiber diameter. This diagram indicates that PCL concentration is the most significant factor, observing how as the concentration increases, so does the fiber diameter. The same occurs with flow rate, while voltage and distance have an inversely proportional influence. However, the presence or absence of the inhibitor does not show a significant effect on the fiber diameter, as mentioned. Finally, the estimated surface response of the significant factors in the average fiber diameter is presented in Supplementary Materials (see Figure S1).

The R^2^ adjusted statistic obtained in the fitting of the model is 94.59% and the model equation is shown below:

**Fiber diameter =** −18.5211 + 3.87059*A + 0.156687*B − 0.0150867*C + 0.00124916*D − 0.286544*E − 0.147281*A^2 − 0.0129375*A*B − 0.02825*A*C + 0.0000260417*A*D + 0.0196875*A*E − 0.00296496*B^2 + 0.0021*B*C − 0.00000333333*B*D − 0.016875*B*E + 0.00574015*C^2 − 0.00001875*C*D + 0.0305*C*E − 2.61456E-7*D^2 − 0.00005625*D*E

### 3.2. Wettability Properties

From an initial point of view, one of the main reasons for the selection of the PCL is associated to the design of highly hydrophobic surfaces [35,66,67]. As has been previously demonstrated, PCL fiber mats with different morphologies have been obtained, including beaded fibers and bead-free fibers as well as variations in fiber diameter, as a function of the variation in the solution concentration and operating parameters during the electrospinning process. For a better comparative study, new electrospun samples have been fabricated in order to have better knowledge about the influence of these parameters (PCL concentration, ZnO NPs and flow rate) in the water contact angle (WCA) measurements at a fixed distance (10 cm) and applied voltage (17.5 kV).

Table 4 presents a summary of experimental conditions with the resultant fiber diameters as well as WCA values of these samples of study with the corresponding presence or absence of beads in the outer surface morphology.

**Figure 8:**
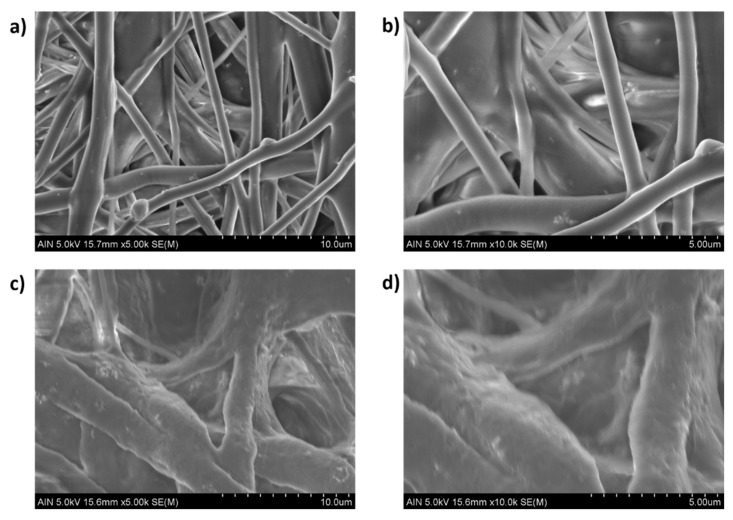
Scanning electron microscopy (SEM) of the electrospun fiber mats at different scale bars (10 and 5 µm) for PCL/ZnO electrospun fiber with 3 wt% ZnO concentration for samples S19 (**a**,**b**) and S18 (**c**,**d**), respectively.

After observing the experimental results derived from Table 4, several conclusions can be obtained. The first is that the addition of 3 wt% ZnO NPs into PCL solution has produced a decrease in the WCA value, which means a more hydrophilic surface [68,69]. It is important that the contact angle directly depends on chemical composition as well as solid–liquid interactions. According to this, neat PCL fibers show a typical hydrophobic nature which is confirmed by a higher water contact angle (WCA) value [70]. However, the presence of surface polar groups in the PCL/ZnO fibers clearly induces a decrease in the resultant water contact angle due to a higher interaction between the surface and the polar solvent (water) [71]. Due to this, the PCL/ZnO samples have shown a better water wettability with a corresponding hydrophobicity reduction. This phenomenon is clearly in the solution composed of 12 wt% PCL where there is change from 126.59 ± 3.71° (S16 without ZnO NPs) up to 104.92 ± 2.21° (S18 with ZnO NPs) for a flow rate of 1000 µL/h, or from 122.71 ± 4.66° (S17 without ZnO NPs) up to 109.22 ± 3.21° (S19 with ZnO NPs) for a flow rate of 2200 µL/h, respectively. In addition, after observing SEM images in Figure 8, this effect of the addition of a high amount of ZnO (3 wt%) can be also associated to the apparition of some specific special morphological features such as the formation of secondary pores in the individual fibers [62] or by the formation of electrospun fibers with a higher degree of porosity [72].

The second one is that the contact angle values for beaded fibers samples (8 wt% PCL) are higher than bead-free fibers samples (12 wt% PCL) [73], the contact angle values being higher for the samples with lower than average fiber diameter. Another important conclusion is that the thinner beaded fibers showed a higher surface roughness and, therefore, the samples showed higher water contact angle values than the thicker bead-free fibers, respectively [74,75]. This effect associated to the design of a multilevel fiber-bead structure has been reported [76] with the aim of obtaining a higher surface roughness, demonstrating that a good control over the operational parameters such a low applied voltage and a high flow rate produces an increase of the resultant surface roughness.

**Figure 9:**
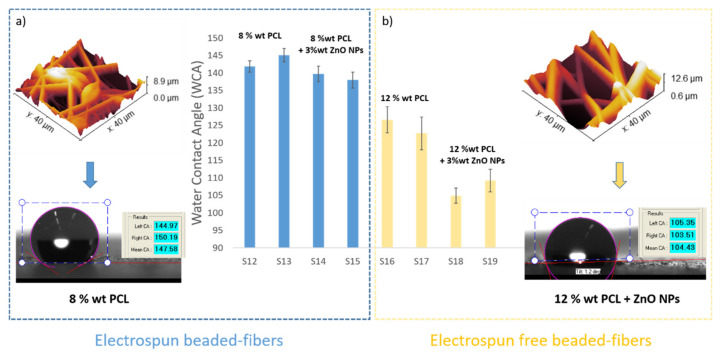
Evolution of the water contact angle (WCA) values for the as-electrospun samples for 8 wt% PCL (**a**) and 12 wt% PCL (**b**) as a function of the presence of electrospun beaded or bead-free fibers, showing the shape of the drop with their corresponding WCA value.

A schematic evolution of the WCA values as a function of the PCL concentration as well as the presence of beads and ZnO NPs can be clearly observed in Figure 9.

**Figure 10:**
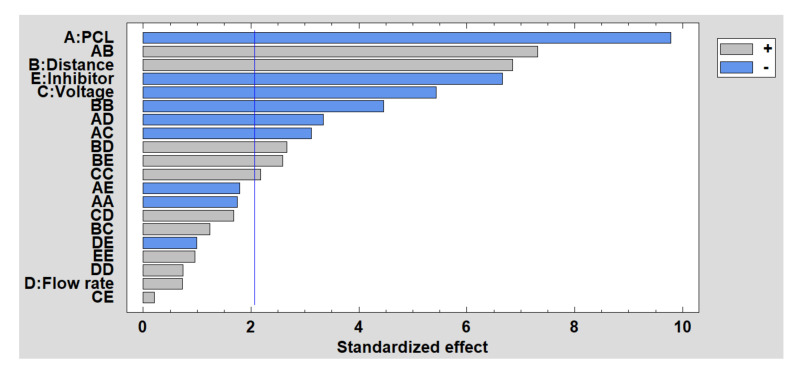
Standardized Pareto chart for contact angle.

Once the results of the experimental tests have been given, the DoE results are shown below to analyze the influence of these factors on wettability, specifically analyzing the water contact angle and the roughness. In Figure 10, the Pareto chart for the contact angle is shown, and it can be observed how the PCL concentration is the most influential factor, so that the higher the concentration is, the smaller the contact angle is. The same happens with the presence of the inhibitor and the voltage, while the distance has a direct influence on the contact angle. Finally, flow rate appears as a non-significant factor in this study. The estimated surface response of the significant factors in the water contact ange is presented in Supplementary Materials (see Figure S2).

The equation of the fitted model is shown below and the adjusted R^2^ statistic obtained in the analysis is 93.84%:

**Contact Angle** = 192.53 + 15.9295*A + 5.59399*B − 19.7685*C − 0.013297*D − 2.07406*E − 0.847697*A^2 + 0.392813*A*B − 0.33525*A*C − 0.00149323*A*D − 0.479687*A*E − 0.345631*B^2 + 0.05305*B*C + 0.000477917*B*D + 0.27825*B*E + 0.674274*C^2 + 0.000602083*C*D + 0.04325*C*E + 0.00000395615*D^2 − 0.000889583*D*E + 1.85421*E^2

**Figure 11:**
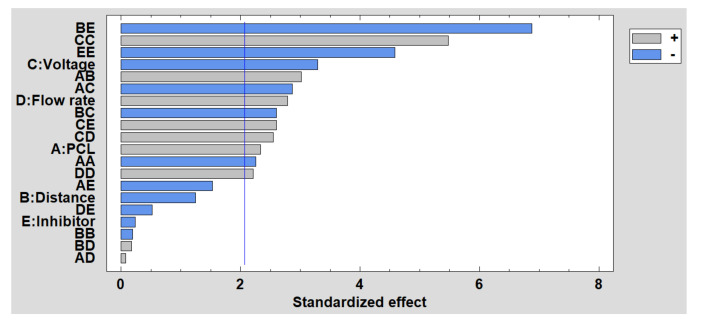
Standardized Pareto chart for the roughness.

In Figure 11 is shown the Pareto diagram for roughness, where the most significant factor is the interaction between distance and the inhibitor. It has been demonstrated that a second porosity thanks to the presence of the inhibitor can induce an increase in the surface roughness, whereas the possibility of obtaining merging fibers with a flattening aspect as a function due to the impact of wet fibers can be produced when the distance is very low to evaporate the solvent. In addition, among the main factors, the most significant is the voltage, so that the higher the voltage, the lower the roughness. However, the opposite effect occurs, as happens with the flow rate and the concentration of PCL. Finally, the estimated surface response of the significant factors in the roughness is presented in Supplementary Materials (see Figure S3).

For the roughness, the mathematical model is defined below and, the adjusted R^2^ statistic obtained in the analysis is 87.67%.

**Roughness** = 20.9114 + 1.69078*A + 0.0216376*B − 3.32244*C − 0.00344861*D + 0.511679*E − 0.0751076*A^2 + 0.0111562*A*B − 0.0211875*A*C + 0.00000234375*A*D − 0.0282812*A*E − 0.00101721*B^2 − 0.007675*B*C + 0.0000021875*B*D − 0.0508125*B*E + 0.116731*C^2 + 0.0000627083*C*D + 0.038375*C*E + 8.18249E-7*D^2 − 0.0000317708*D*E − 0.61043*E^2

### 3.3. Corrosion Resistance

First of all, in order to corroborate a relationship between the resultant wettability and the corrosion behavior of the electrospun samples, the corrosion resistance has been determined by using potentiodynamic polarization curves in a chloride corrosive solution (3.5 wt% NaCl). Previous works have demonstrated that the use of electrospun fibers with an intrinsic hydrophobic behavior can produce an enhancement in the corrosion resistance, being some representative examples the poly(vinylidene fluoride) (PVDF) [77,78], polyaniline [79], poly-(vinyl chloride) (PVC) [51], polystyrene (PS) [80], polycaprolactone (PCL) [81] or perfluorinated block polymers [82], among others. Another aspect to remark upon is that the combination of metallic oxides which act as corrosion inhibitors with electrospun fibers with this hydrophobic behavior have also been employed as a novel strategy for the development of highly anti-corrosive surfaces [83]. In this sense, electrospun fiber mats act as ideal hosts for the corrosion inhibitors, being a representative example the use of ZnO nanoparticles. A significant example is presented in [84] where PVDF-ZnO nanocomposite coating has been deposited onto aluminum, showing significantly less susceptibility to corrosion in comparison with pure PVDF. More specifically, the protection efficiency has been increased from 84% (PVDF) up to 99% (PVDF-ZnO). In addition, another important advantage is that ZnO NPs can be perfectly distributed without using any dispersing agent in only one-step fabrication [85]. Another representative example is presented in [86] where a comparative study in terms of corrosion resistance is performed by using polymeric fibers of polystyrene (PS) and polyvinyl chloride (PVC) with incorporated ZnO nanoparticles deposited onto aluminum [86]. It has been demonstrated that the electrospun fibers doped with ZnO NPs have produced an important reduction of the corrosion current of the aluminum substrate in two orders of magnitude, showing also an important enhancement against pitting corrosion resistance. Other representative works by using PCL-ZnO NPs deposited onto magnesium [68] or a combination of metallic oxides (PCL/ZnO-NiO-CuO) deposited onto mild steel [87] have also been implemented, and in both cases also endow an increase in the corrosion resistance. Our experimental results also corroborate that the electrospun samples containing ZnO NPs showed better corrosion properties because the resultant corrosion rate (mm/year) is decreased in one order of magnitude in comparison with the free ZnO NPs electrospun fibers, indicating a better corrosion protection efficiency, as can be appreciated in Table 5.

**Figure 12:**
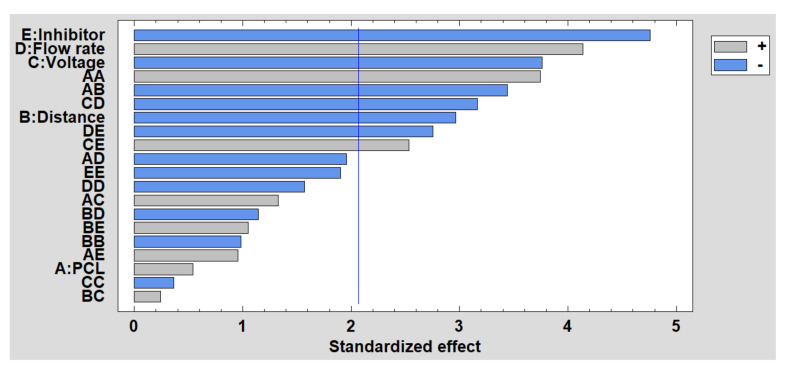
Standardized Pareto chart for corrosion resistance.

In order to complement the corrosion analysis, the results obtained in the DoE are shown below. Figure 12 shows the Pareto diagram, and it can be clearly observed that the presence of the inhibitor is the most significant factor because less ZnO NPs induces a higher corrosion rate. This reverse effect also occurs with voltage and distance, while the effect of flow rate is the opposite because a higher flow rate induces a higher corrosion rate. Finally, the estimated surface response of the significant factors in the corrosion rate is presented in Supplementary Materials (see Figure S4).

For the corrosion rate, the adjusted R^2^ statistic obtained in the analysis is 84.74% and the mathematical fitted model is shown below:

**Corrosion rate** = 0.135041 − 0.0554376*A + 0.00650306*B + 0.00457974*C + 0.0000966358*D − 0.0178052*E + 0.00294862*A^2 − 0.000300614*A*B + 0.000232157*A*C − 0.0000014242*A*D + 0.00041762*A*E − 0.000123936*B^2 + 0.0000167768*B*C − 3.32823E-7*B*D + 0.000183206*B*E − 0.000182681*C^2 − 0.00000184275*C*D + 0.000884631*C*E − 1.37228E-8*D^2 − 0.00000401286*D*E − 0.0059912*E^2.

## 4. Conclusions

In this work, multifunctional coatings with a desired morphology in terms of fiber diameter, wettability and anti-corrosion properties have been analyzed in depth by using the electrospinning technique. In this sense, PCL has been employed as the polymeric precursor for the fabrication of the electrospun fibers with the presence or absence of beads as a function of the solution concentration, whereas ZnO NPs have been used for an enhancement in the corrosion resistance with a change in the wettability properties. In order to assess these influential effects, a DoE has been performed as a function of five experimental variables such as the PCL concentration, flow rate, applied voltage, tip-collector distance and presence/absence of ZnO NPs, analyzing their corresponding effects in the fiber diameter, water contact angle value, roughness and corrosion rate, respectively. An initial conclusion is that PCL concentration is the most influential factor for the fiber diameter and water contact angle. According to this, an increase in the PCL concentration enables an increase in the fiber diameter, whereas the water contact angle is gradually decreased. This effect is associated with the presence of beaded-fibers at the lowest PCL concentration. In addition, the interaction between the distance and the inhibitor as well as the presence of ZnO NPs are the most significant factors for the resultant roughness. Finally, for the corrosion rate, the most significant factor is the amount of ZnO NPs where it is corroborated that the electrospun fiber coatings with a higher amount of ZnO has produced a better corrosion protection efficiency.

## Figures and Tables

**Table 1:** Summary of all the electrospun coatings as a function of the nature of fluid (PCL concentration and dispersed ZnO NPs) and the operational parameters (flow rate, applied voltage and distance to collector).

PCL	ZnO NPs	Flow Rate	Applied Voltage	Distance
8, 10, 12 wt%	0, 1, 3 wt%	1, 1.6, 2.2 mL/h	12.5, 15, 17.5 kV	10, 15, 20 cm

**Table 2:** Design of experiment (DoE) with the factors evaluated with their corresponding high and low levels.

Factor	High Level	Low Level
(A) PCL concentration (%)	12	8
(B) Distance (cm)	20	10
(C) Voltage (kV)	17.5	12.5
(D) Flow rate (µL/h)	2200	1000
(E) Inhibitor presence	1	−1

**Table 3:** Summary of the electrospun coatings fabricated as a function of the PCL concentration, ZnO NPs concentration, distance, applied voltage and flow rate, respectively.

Number of Sample	PCL (wt%)	ZnO NPs (wt%)	Distance (cm)	Voltage (kV)	Flow Rate (µL/h)	Fiber Diameter (µm)
S1	10	1	15	15	1000	3.21 ± 0.11
S2	10	1	15	12.5	1600	4.21 ± 0.13
S3	10	1	15	15	1600	3.66 ± 0.21
S4	10	1	15	17.5	1600	3.18 ± 0.11
S5	10	1	15	15	2200	4.02 ± 0.13
S6	10	1	10	15	1600	3.95 ± 0.32
S7	10	1	20	15	1600	3.32 ± 0.07
S8	10	0	15	15	1600	3.68 ± 0.16
S9	10	3	15	15	1600	3.71 ± 0.11
S10	8	1	15	15	1600	2.13 ± 0.09
S11	12	1	15	15	1600	4.11 ± 0.08

**Table 4:** Summary of the electrospun coatings fabricated as a function of the PCL concentration, ZnO NPs concentration and flow rate with their corresponding fiber diameter, WCA values and presence/absence of beads in the surface morphology.

Number of Sample	PCL (wt%)	ZnO NPs (wt%)	Flow Rate (µL/h)	Roughness Sa (µm)	Fiber Diameter (µm)	WCA (°)	Presence of Beads
S12	8	0	1000	1.76 ± 0.10	2.11 ± 0.28	141.86 ± 1.61	Ok
S13	8	0	2200	1.88 ± 0.09	2.35 ± 0.15	145.09 ± 1.90	Ok
S14	8	3	1000	2.09 ± 0.04	2.04 ± 0.21	139.74 ± 2.21	Ok
S15	8	3	2200	2.02 ± 0.10	2.44 ± 0.19	137.98 ± 2.29	Ok
S16	12	0	1000	1.49 ± 0.03	3.03 ± 0.51	126.59 ± 3.71	No
S17	12	0	2200	1.74 ± 0.04	3.48 ± 0.44	122.71 ± 4.66	No
S18	12	3	1000	1.67 ± 0.14	3.70 ± 0.44	104.92 ± 2.21	No
S19	12	3	2200	1.58 ± 0.13	4.04 ± 0.29	109.22 ± 3.21	No

**Table 5:** The electrochemical parameters from Tafel polarization curves for bare stainless steel (AISI 304) and the PCL electrospun samples after immersion in 3.5 wt% NaCl aqueous solution.

Sample	β_a_ (mV/dec)	β_c_ (mV/dec)	E_corr_ (V)	*i*_corr_ (A)	Corrosion Rate (mm/Year)
Reference steel	0.19443	0.2284	−0.10592	1.91E-05	0.011831
S12	0.6528	0.15769	−0.27102	1.4094E-06	0.0073271
S13	0.40458	0.079877	−0.20795	1.1164E-06	0.0058039
S14	0.15224	0.048841	−0.079322	3.1208E-08	0.00016224
S15	0.17886	0.089351	−0.30841	2.1332E-06	0.0013112
S16	0.24296	0.13855	−0.27897	3.0928E-06	0.009079
S17	0.72226	0.15059	−0.22431	1.484E-06	0.0061553
S18	0.41468	0.097008	−0.28954	2.1699E-06	0.0013338
S19	0.51866	0.24543	−0.34638	1.0355E-07	0.00095943

## Supplementary Materials

The following are available online at https://www.mdpi.com/article/10.3390/polym13244312/s1. Figure S1: Estimated surface response of significant factors in the average fiber diameter, Figure S2: Estimated surface response of significant factors in the water contact angle, Figure S3: Estimated surface response of significant factors in the roughness, Figure S4: Estimated surface response of significant factors in the corrosion rate.
