# Supplementary material for: Modeling Experimental Parameters for the Fabrication of Multifunctional Surfaces Composed of Electrospun PCL/ZnO-NPs Nanofibers
Source: Polymers (Basel). 2021 Dec 9;13(24):4312. doi: 10.3390/polym13244312 (PMC8706923; doi:10.3390/polym13244312)
Supplement: Supplementary file 1 [file polymers-13-04312-s001.zip › polymers-1462524-supplementary.pdf]

Supplementary Materials

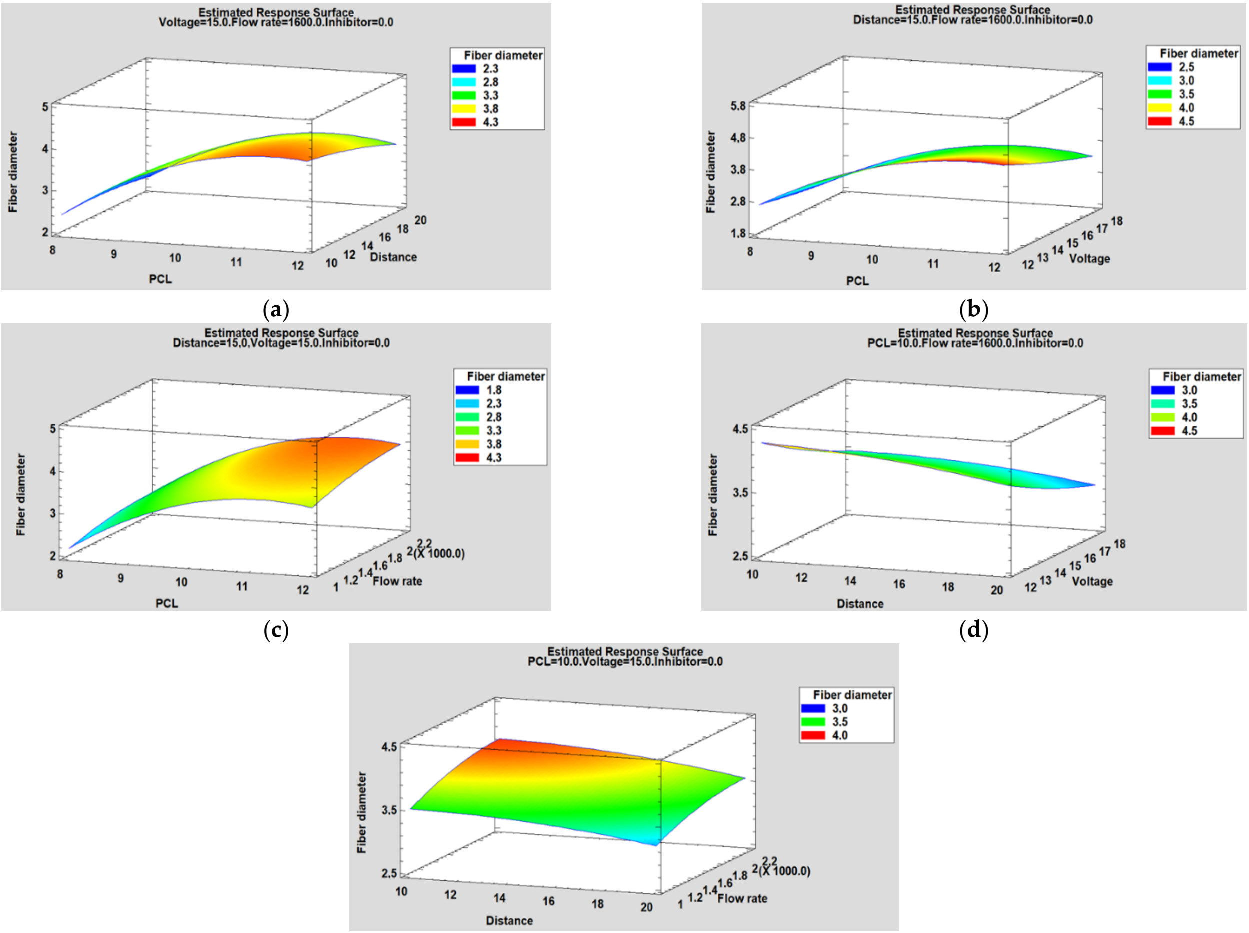

(**a**) (**b**) (**c**) (**d**) (**e**)

Figure S1. Estimated surface response of significant factors in the average fiber diameter. (**a**) voltage, flow rate and inhibitor response surface, (**b**) distance, flow rate and inhibitor response surface, (**c**) distance, voltage and inhibitor response surface, (**d**) PCL, flow rate and inhibitor response surface, (**e**) PCL, voltage and inhibitor response surface.

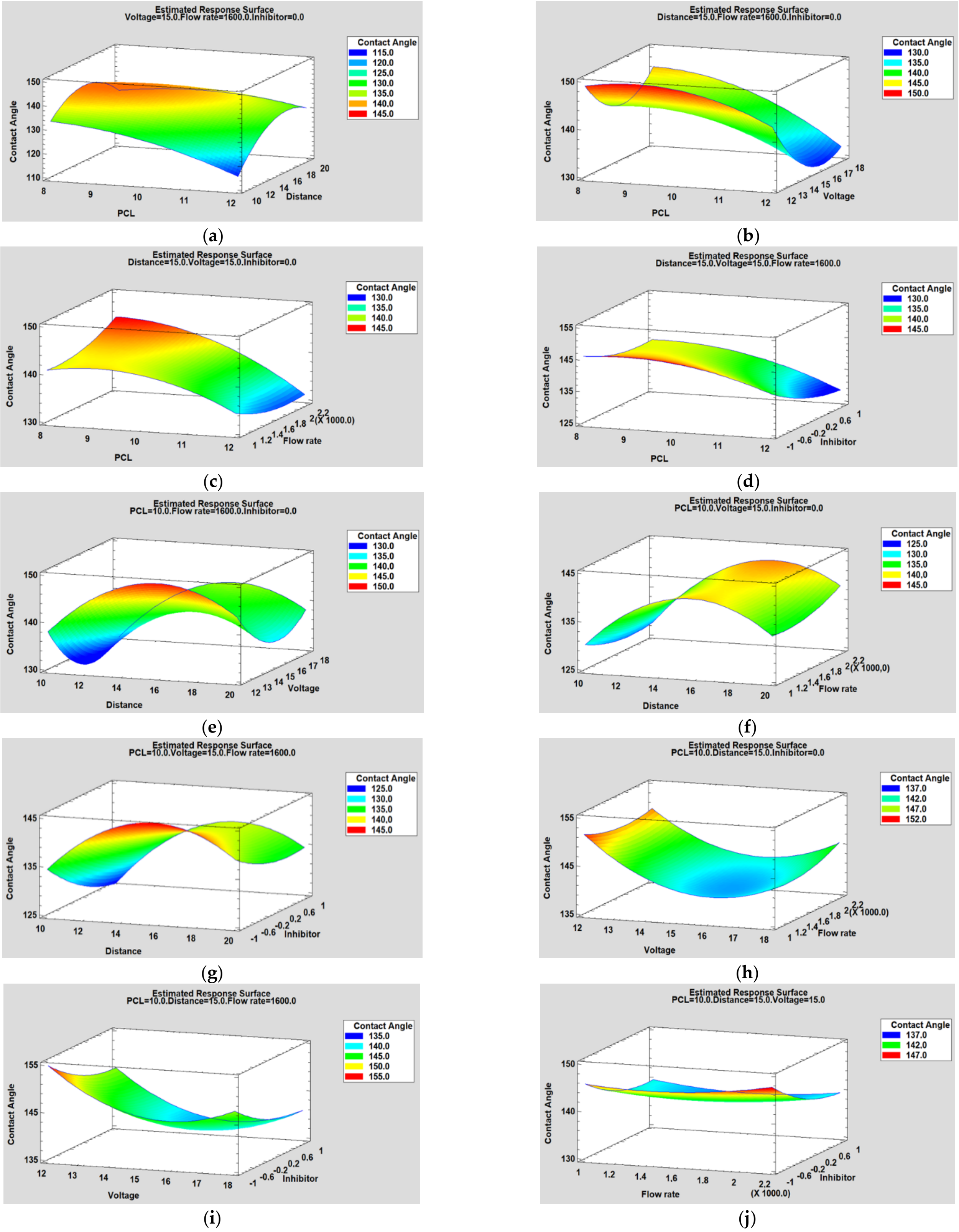

Figure S2. Estimated surface response of significant factors in the water contact angle. (**a**) voltage, flow rate and inhibitor response surface, (**b**) distance, flow rate and inhibitor response surface, (**c**) distance, voltage and inhibitor response surface, (**d**) distance, voltage and flow rate response surface, (**e**) PCL, flow rate and inhibitor response surface, (**f**) PCL, voltage and inhibitor response surface, (**g**) PCL, voltage and flow rate response surface, (**h**) PCL, distance and inhibitor response surface, (**i**) PCL, distance and flow rate response surface, (**j**) PCL, distance and voltage response surface.

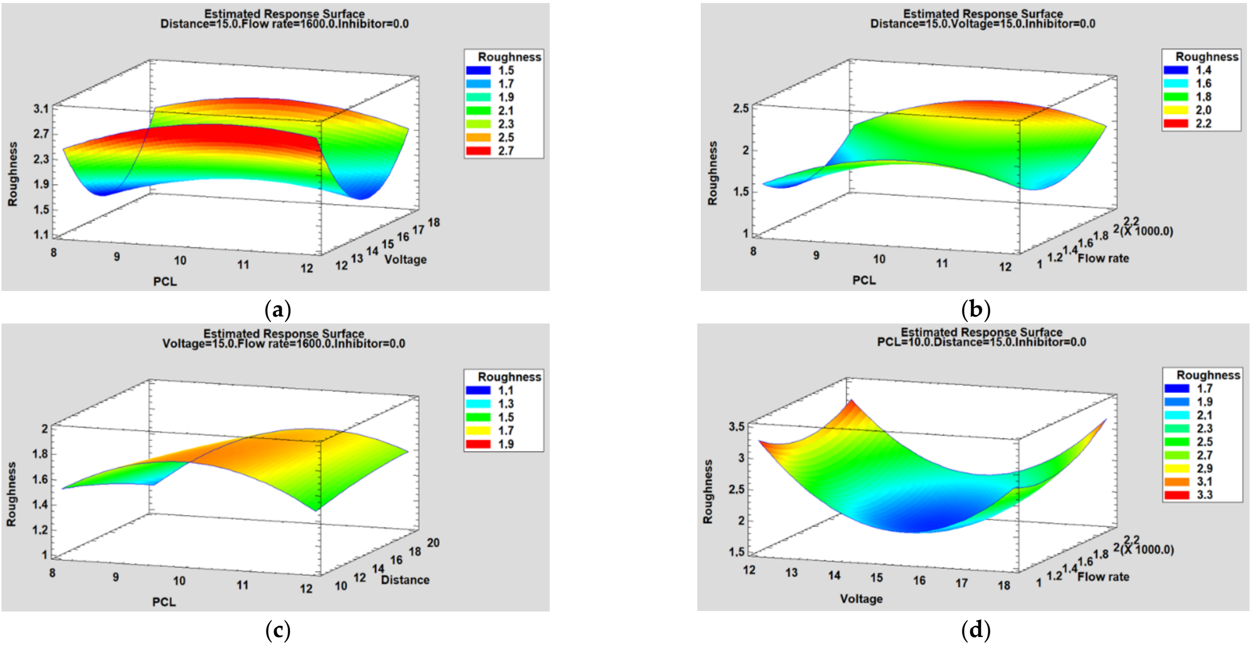

Figure S3. Estimated surface response of significant factors in the roughness. (a) distance, flow rate and inhibitor response surface, (b) distance, voltage and inhibitor response surface, (c) voltage, flow rate and inhibitor response surface, (d) PCL, distance and inhibitor response surface.

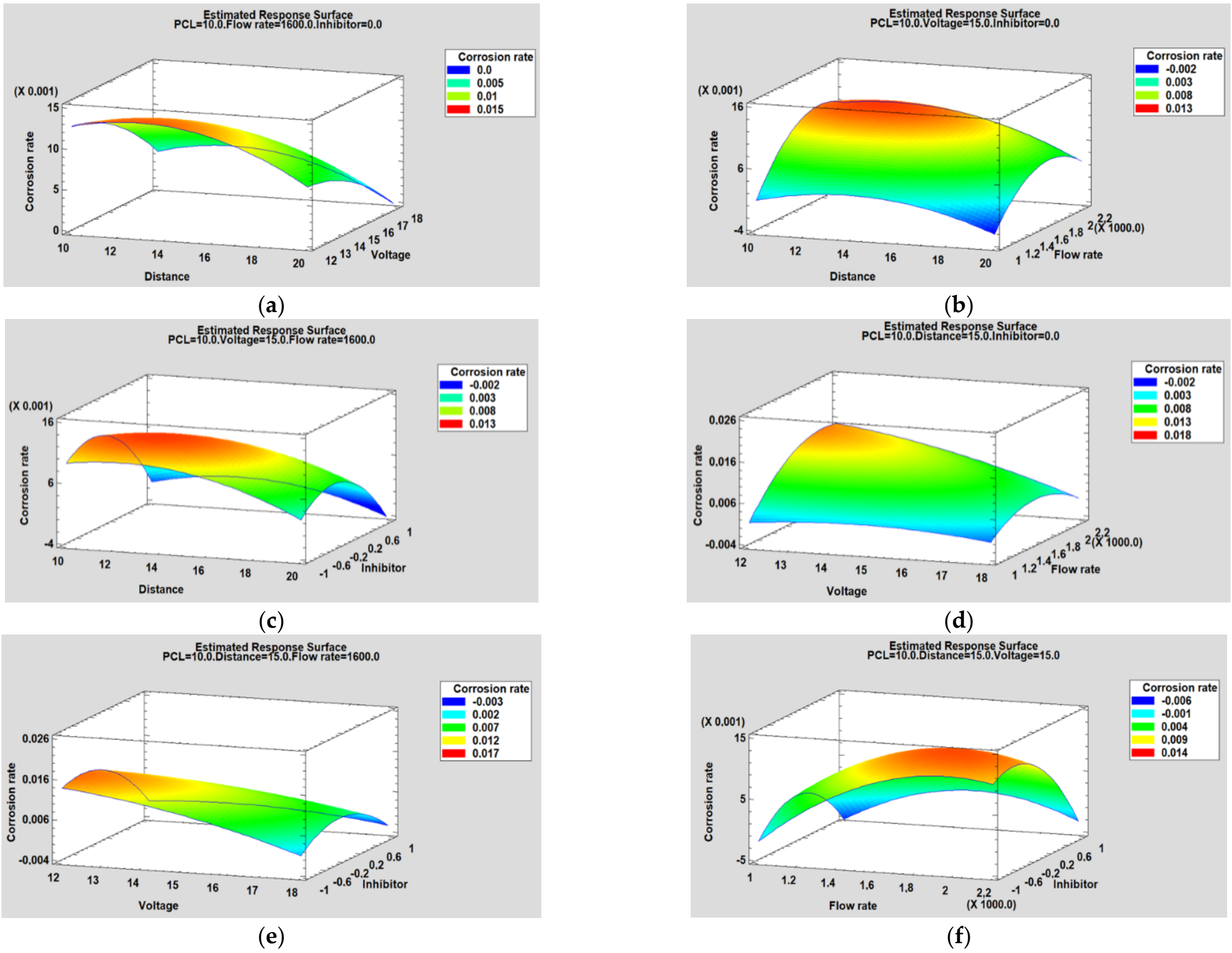

Figure S4. Estimated surface response of significant factors in the corrosion rate. (**a**) PCL, flow rate and inhibitor response surface, (**b**) PCL, voltage and inhibitor response surface, (**c**) PCL, voltage and flow rate response surface, (**d**) PCL, distance and inhibitor response surface, (**e**) PCL, distance and flow rate response surface, (**f**) PCL, distance and voltage response surface.
